# Editorial: Special Issue “Unmanned Aerial Vehicle (UAV)-Enabled Wireless Communications and Networking”

**DOI:** 10.3390/s22124458

**Published:** 2022-06-13

**Authors:** Margot Deruyck

**Affiliations:** Department of Information Technology, IMEC-Ghent University-WAVES, 9052 Ghent, Belgium; margot.deruyck@ugent.be

In the last decade, the behavior of mobile data users has completely changed. We are no longer limited to our own house if we want to share pictures, make a video call, stream music or video, or game. In fact, nowadays, we can do all those things in the park, bus, or train, actually wherever we are. With the upcoming next generation standard—the 5th Generation or 5G—not only will significantly higher data rates will be offered, but also an ever larger spectrum of applications and services to the mobile users and platforms, all with heterogeneous requirements. Besides serving users, 5G will also bring many opportunities to the industry. This means that 5G will be serving a massive density of nodes which can either be human-held or machine-type; a tendency we currently already see in the emerging Internet of Things (IoT) and its applications. The above-mentioned evolution will of course also have its repercussions on the network itself. The future wireless network will be characterized by a high degree of flexibility compared with the past, allowing them to adapt smoothly, autonomously, and efficiently to the quickly changing traffic demand evolutions both in time and space. The networks of today are designed to cope with average or peak traffic predictions and the offered capacity on a local scale is highly dependent on the density of the infrastructure equipment in a considered area. Adding mobile infrastructure or UABSs (Unmanned Aerial Base Stations)—i.e., a base station mounted on a UAV (Unmanned Aerial Vehicle) or drone—would provide a huge added value to the network.

One of the key challenge of designing such a UAV-based network is a proper allocation of both the UAV’s location and network resources. Ref. [1] proposed a fast and practical algorithm for UAV relay networks to provide the optimal solution for the number of transmit time slots and the UAV relay location in a sequential manner. The transmit power at the base station and the UAV was determined in advanced based on the availability of channel state information (CSI). Simulation results demonstrated that the proposed algorithms can significantly reduce the computational effort and complexity to determine the optimal UAV location. Ref. [2] formulated the uplink resource allocation problem for a NOMA (Non-Orthogonal Multiple Access)-IoT based UAV network with the objective of maximizing the system throughput while minimizing the delay of IoT applications. Power allocation was investigated to achieve fairness between the users. The algorithm achieved a 31.8% delay improvement, 99.7% reliability increase, and 50.8% fairness enhancement when compared with the maximum channel quality indicator CQI) algorithm, allowing the algorithm to be used for URLLC (Ultra-Reliable Low-Latency Communication) applications. Due to the large-scale deployment of UAVs in UAV-based networks, also the shortage of spectrum is a realistic threat for the roll-out of these networks. Ref. [3] proposed a cooperative spectrum sensing scheme for cognitive UAV networks based on a CHMM (Continuous Hidden Markov Model) with a novel SNR (Signal-to-Noise Ratio) estimation method.

Another typical use case for UAV-aided networks—besides offering an extra degree of flexibility in the network—is to extend the network’s coverage in isolated or rural areas. Ref. [4] comprehensively surveyed the user of three platforms to deliver broadband services to remote and low-income areas: UAVs, APs (Altitude Platforms), and LEO (Low Earth Orbit) satellites. The UAVs are considered as a noteworthy solution since their efficient maneuverability can solve rural coverage issues or not-spots. Ref. [5] investigated six different routing algorithms for this kind of applications. A time-dependent variant of the Dijkstra’s algorithm which determines the fastest route by taking into account the time when the message reaches the node and the time allocated for data transfer was developed for a DTN (Delay Tolerant Network) of drones flying in an aerial area divided either into squares or into equilateral triangles. Their simulation results showed a better performance of the proposed time-dependent Dijkstra algorithm compared with state-of-the art routing protocols. One step further than extending the coverage of an existing network is to provide wireless communication in areas hit by a natural disaster. Ref. [6] jointly addressed the transmission power selection, data-rate maximization, and interference mitigation problems for such a scenario. Considering all these conflicting parameters, the problem was investigated as a budget-constrained multi-player multi-armed bandit problem. The whole process was carried out in a decentralized manner, where no information was exchanged between UAVs. To achieve this, two power-budget-aware algorithms were proposed to realize the selection of the transmission power value efficiently. Both algorithms showed outstanding performance over random power value selection in terms of achievable data rate. UAVs have also showed their added value for the enhancement of wireless communication in millimeter-wave bands. Typically, antenna arrays have been employed for this purpose. However, many beam-forming methods for improving communication quality are based on channel estimation, which are resource-intensive due to the complexity of channel estimation in practice. Ref. [7] formulated a MIMO (Massive Input Massive Output) blind beamforming problem at the receivers for UAV-assisted communications in which channel estimation was omitted to save resources. An analytical method, called ACMA (Analytical Constant Modulus Algorithm), was introduced relying only on data received by the antenna. ACMA could achieve good signal recovery accuracy, a reasonable sum rate, and acceptable complexity.

Many challenges related to the design of UAV networks in general, such as, for example, spectrum sharing/coexistence, are computationally very challenging and hence an excellent case to apply machine learning and AI (Artificial Intelligence) on. Ref. [8] proposed a multi-agent DQL (Deep Q-learning)-based transmission power control algorithm to minimize the outage probability while satisfying the interference requirement of an interfered system. To deal with the potential risk of action-value overestimation from the DQL, they developed even a DDQL (Double DQL). The proposed DQL power control algorithm performed equal or close to the optimal exhaustive search algorithm for varying positions of the interfered system. With a similar performance by the DDQL power control yields the same performance, the authors concluded that the actional value overestimation did not adversely affect the quality of the learned power control policy. Besides using machine learning to actual develop the network, it can also be applied in properly predicting the user demand. Ref. [9] proposed a UAV positioning algorithm with the objective of extending an existing network and balancing the traffic load. To properly predict the users and their movement, the performance of various state-of-the-art machine learning algorithms was investigated. Random Forest and Gradient Boosting presented both the best performance, with Random Forest having a better prediction and training time.

The use of UAVs in wireless communication networks—whether it is to provide extra flexibility, extend the coverage or cover isolated, rural, or post-disasters areas—makes the network more vulnerable for security issues. Ref. [10] proposed a security model applying cooperative friendly jamming using artificial noise and drone mobility to prevent eavesdropping and improve security. Ref. [11] developed two dynamic selection techniques which identify the most effective classifier for the detection of GPS (Global Positioning System) spoofing attacks.

It might be clear that using UAVs for wireless communication and networks can provide a tremendous added value. The scenarios here presented are only a fraction of what we can achieve by UAV-aided communication. A nice overview of what is possible was provided in ref. [12], along with the recent advances and challenges that are brought by the recent research trends.
